# Effects of low-dose/high-dose-rate X-irradiation on oxidative stress in organs following forced swim test and its combined effects on alcohol-induced liver damage in mice

**DOI:** 10.1093/jrr/rrad030

**Published:** 2023-05-17

**Authors:** Shota Naoe, Yuki Fujimoto, Kaito Murakami, Ryohei Yukimine, Ayumi Tanaka, Kiyonori Yamaoka, Takahiro Kataoka

**Affiliations:** Graduate School of Health Sciences, Okayama University, 5-1 Shikata-cho 2-chome, Kita-ku, Okayama-shi, Okayama 700-8558, Japan; Graduate School of Health Sciences, Okayama University, 5-1 Shikata-cho 2-chome, Kita-ku, Okayama-shi, Okayama 700-8558, Japan; Graduate School of Health Sciences, Okayama University, 5-1 Shikata-cho 2-chome, Kita-ku, Okayama-shi, Okayama 700-8558, Japan; Graduate School of Health Sciences, Okayama University, 5-1 Shikata-cho 2-chome, Kita-ku, Okayama-shi, Okayama 700-8558, Japan; Graduate School of Health Sciences, Okayama University, 5-1 Shikata-cho 2-chome, Kita-ku, Okayama-shi, Okayama 700-8558, Japan; Faculty of Health Sciences, Okayama University, 5-1 Shikata-cho 2-chome, Kita-ku, Okayama-shi, Okayama 700-8558, Japan; Faculty of Health Sciences, Okayama University, 5-1 Shikata-cho 2-chome, Kita-ku, Okayama-shi, Okayama 700-8558, Japan

**Keywords:** low-dose/high-dose-rate irradiation, forced swim test, alcohol, oxidative stress, antioxidants

## Abstract

The liver’s susceptibility to oxidative stress after a combination of forced swim test (FST) and low-dose-rate γ-irradiation has been observed. Therefore, this study aims to clarify the effects of low-dose (0.1 and 0.5 Gy)/high-dose-rate (1.2 Gy/min) irradiation on combined oxidative stressors—liver damage associated with FST and alcohol administration. In addition, the effects of similar irradiation on FST-induced immobility, which induces psychomotor retardation, and antioxidative effects on the brain, lungs, liver and kidneys were investigated, and the results were compared with those of a similar previous study that utilized low-dose-rate irradiation. Low-dose/high-dose-rate (especially 0.5 Gy) irradiation temporarily worsened liver antioxidant function and hepatic function with FST- and alcohol administration-related oxidative damage; however, the damages improved soon after. In addition, the increase in total glutathione content in the liver contributed to the early improvement of hepatic functions. However, pre-irradiation did not suppress immobility during the FST. The results also suggested that the effects of low-dose/high-dose-rate irradiation on the antioxidant functions of each organ after the FST were different from those of low-dose/low-dose-rate irradiation. Overall, this study provides further insights into the effects of low-dose irradiation on exposure to a combination of different oxidative stressors. It will also contribute to the elucidation of dose rate effects on oxidative stress in the low-dose irradiation range.

## INTRODUCTION

We have previously reported that low-dose irradiation enhances antioxidative function in various mice organs and suppresses oxidative stress-induced diseases [1]. For example, it was revealed that cold-induced brain injury was inhibited by increasing superoxide dismutase (SOD) activity and decreasing lipid peroxide (LPO) levels in mouse brains upon 0.5 Gy X-ray irradiation [2]. We have also reported that 0.5 Gy X-ray [3] and γ-ray irradiation [4] suppressed carbon tetrachloride-induced liver injury in mice by increasing SOD and catalase (CAT) activities, as well as total glutathione (t-GSH) content, respectively. Moreover, 0.5 Gy γ-irradiation enhanced SOD activity in the pancreas and suppressed type I diabetes in mice [5].

The forced swim test (FST) has been reported to increase rodent oxidative stress and induce immobility, which causes psychomotor retardation [6, 7]. Repeated FSTs also cause oxidative stress in the brain as well as tissue degeneration and oxidative stress in peripheral tissues, including the liver, which is regulated by the brain [6, 7]. However, it was revealed that low-dose (0.5 Gy) and low-dose-rate (3.0 mGy/h) γ-irradiation increased CAT activity in the mouse brain and inhibited FST-associated immobility. In addition, the effects of low-dose-rate γ-ray irradiation on antioxidant function following FST varied depending on the organs [8]. SOD activity and t-GSH content in the liver of 3.0-mGy/h irradiated mice followed by FST were significantly lower than those in the control mice. Although there were no significant differences in antioxidative functions in the kidneys of the FST group and FST with irradiation group animals, CAT activity in the lungs of 3.0-mGy/h γ-irradiated mice was higher than that in the control and FST mice [8]. These findings indicate that the liver may be susceptible to oxidative stress following a combination of FST and low-dose irradiation. Similar effects have been reported for oxidative stress inhibition using low-dose/high-dose-rate and low-dose/low-dose-rate irradiation. However, the differences between low-dose/high-dose-rate and low-dose/low-dose-rate irradiation regarding inhibition of oxidative stress are yet to be reported.

Based on the above, the combination of low-dose and low-dose-rate irradiation and FST caused oxidative stress on the liver, which would be expected to develop into further liver injury when coupled with the administration of alcohol. In addition, although low-dose irradiation has been confirmed to suppress various types of liver damage, the effect of low-dose irradiation on liver damage associated with both FST and alcohol administration is yet to be fully elucidated.

This study aims to elucidate the effects of low-dose/high-dose-rate X-irradiation on combined oxidative stress exposure. First, since it was well established that alcohol administration induces oxidative damage in the liver [9–12], the effects of irradiation on liver injury associated with combined FST and alcohol administration were investigated using prior low-dose/high-dose-rate irradiation. Second, the immobile state of mice during the FST and the antioxidant function in each organ due to the low-dose irradiation were investigated, and the results were compared with those of our previous study using low-dose/low-dose-rate irradiation [8].

## MATERIALS AND METHODS

### Animals

BALB/c mice (8-week-old males) were sourced from Jackson Laboratory Japan Inc. (Yokohama, Japan). All study-related protocols and experiments were approved by the Animal Care and Use Committee of Okayama University. The mice were housed under a half-day light cycle at a temperature of 22 ± 2°C and had free access to food and water.

### Experimental procedure

**Fig. 1:**
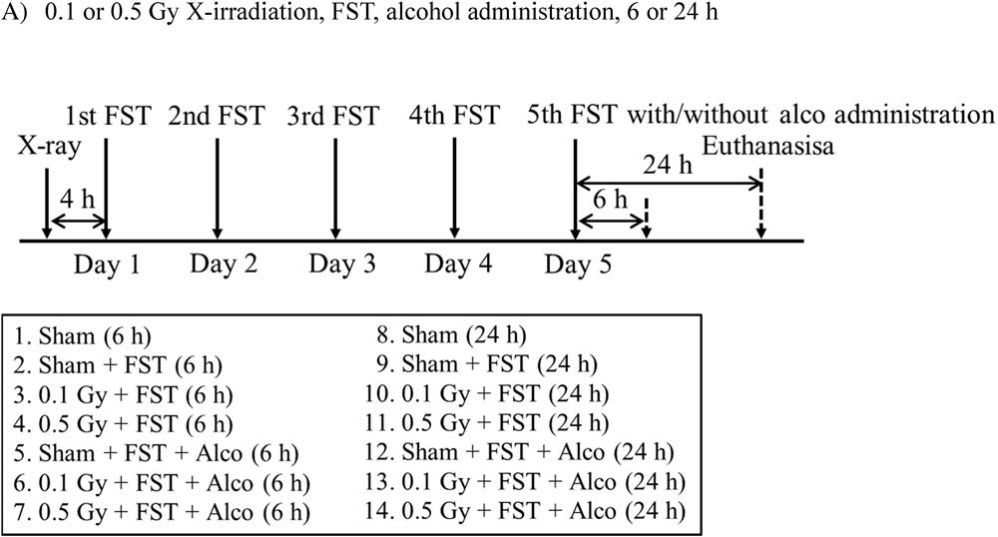
Experimental procedure: prior 0.1 or 0.5 Gy X-irradiation and alcohol administration at 6 or 24 h after FST.

The mice were divided into 14 groups (4–7 mice in each group) (Fig. 1), 7 groups at 6 h after treatment (Fig. 1, 1–7): Sham irradiation (Sham [6 h] group), Sham irradiation with FST (Sham + FST [6 h] group), 0.1 Gy X-irradiation with FST (0.1 Gy + FST [6 h] group), 0.5 Gy X-irradiation with FST (0.5 Gy + FST [6 h] group), Sham irradiation with FST and alcohol administration (Sham + FST + Alco [6 h] group), 0.1 Gy X-irradiation with FST and alcohol administration (0.1 Gy + FST + Alco [6 h] group) and 0.5 Gy X-irradiation with FST and alcohol administration (0.5 Gy + FST + Alco [6 h] group), and another 7 groups at 24 h after treatment (Fig. 1, 8–14): Sham irradiation (Sham [24 h] group), Sham irradiation with FST (Sham + FST [24 h] group), 0.1 Gy X-irradiation with FST (0.1 Gy + FST [24 h] group), 0.5 Gy X-irradiation with FST (0.5 Gy + FST [24 h] group), Sham irradiation with FST and alcohol administration (Sham + FST + Alco [24 h] group), 0.1 Gy X-irradiation with FST and alcohol administration (0.1 Gy + FST + Alco [24 h] group) and 0.5 Gy X-irradiation with FST and alcohol administration (0.5 Gy + FST + Alco [24 h] group). The mice were subjected to 0.1 or 0.5 Gy X-irradiation and Sham irradiation. Next, FST was performed five times, and alcohol was administered after the final FST. After a period of 6 or 24 h after administration, the mice were euthanized using CO_2_.

### X-ray irradiation

The whole bodies of mice were irradiated. The dose rate was 1.2 Gy/min, and the total doses were 0.1 or 0.5 Gy (tube voltage, 150 kV; tube current, 20 mA; filter, 0.5 mm Al and 0.2 mm Cu; distance between focus and target, 43.5 cm), which were delivered using an X-ray generator (MBR-1520R-3; Hitachi Power Solutions Co., Ltd, Ibaraki, Japan). Control mice were Sham-exposed. During the treatment, all mice were housed in a small cage (fan-shaped; radius 10 cm, height 4.5 cm, angle 30°) and rotated for 24 s. After the target doses were achieved, only the rotation was continued. In the previous study, we examined the effects of 0.1, 0.5, 1.0 and 2.0 Gy (dose rate: 1.2 Gy/min) X-irradiation on five FSTs; however, as 1.0 and 2.0 Gy irradiation exerted little effect on immobility and antioxidant function in the brain [13], these conditions were not utilized in this study.

### FST and alcohol administration

**Fig. 2:**
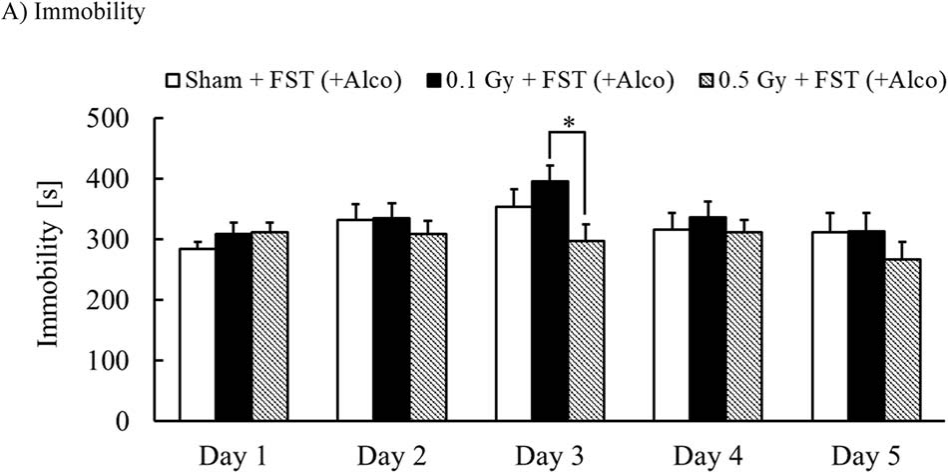
Effects of X-irradiation on FST-induced immobility. Mean ± standard error of the mean, *n* = 14, ^*^P < 0.05.

The FST was performed and repeated daily for 5 days, following a method described by Joram *et al*. [14], similar to the one we previously described [8]. Five observers blinded to the scheme determined the immobility time of the mice. Immobility was defined as the point when the mice ceased struggling and remained motionless, floating in the water and making only movements necessary to keep their heads above the surface of the water. The immobility time was measured for the groups with 6 h to euthanasia but not for those with 24 h to euthanasia, since the difference in time to euthanasia was considered to be ineffective on immobility during the FST period. In addition, the immobility data for the Sham + FST group included the Sham + FST (6 h) and Sham + FST + Alco (6 h) groups, the 0.1 Gy + FST group included the 0.1 Gy + FST (6 h) and 0.1 Gy + FST + Alco (6 h) groups and the 0.5 Gy + FST included the 0.5 Gy + FST (6 h) and 0.5 Gy + FST + Alco (6 h) groups. (Fig. 2) This is because these groups received the same treatments until the final FST.

After the final FST, the mice were intraperitoneally administered 50% ethanol at 5.0 g/kg body weight.

### Sample preparation

At 6 or 24 h after alcohol administration, mice were euthanized using CO_2_; their brain, lungs, liver and kidneys were removed rapidly, and blood was obtained. The serum was separated via centrifugation at 3000 × *g* for 5 min at 4°C. The organs and serum were stored at −80°C until analysis.

### Biochemical assays

Serum glutamic oxaloacetic transaminase (GOT) and glutamic pyruvic transaminase (GPT) activities were analyzed with an assay kit (Wako Pure Chemical Industry, Co., Ltd, Osaka, Japan). Briefly, a mixture of enzymes and a chromogenic solution was incubated at 37°C for 5 min and then reacted with the serum. After incubation at 37°C for 20 min, a reaction-stopping solution was added, and the optical density of the product was measured at 555 nm using a spectrophotometer.

SOD and CAT activities, t-GSH content and total protein levels in the organs were analyzed using assay kits (SOD: Dojindo Molecular Technologies, Inc, Kumamoto, Japan; CAT: Cayman Chemical, MI, USA; t-GSH: OXIS Health Products, Inc, Portland, OR, USA; total protein: Dojindo Molecular Technologies, Inc, Kumamoto, Japan). These were measured following a previously described method [13]. The data are presented as the ratio of each activity value divided by the total protein content, and then further as the ratio to the control.

### Statistical analyses

Data are presented as the mean ± standard error of the mean. The statistical significance of differences was determined using a one-way analysis of variance and Tukey’s test. Differences between groups were considered statistically significant at *P* < 0.05.

## RESULTS

### Effects of X-irradiation on FST-induced immobility

On Day 3, the immobility of 0.1 Gy-irradiated mice was significantly higher than that of 0.5 Gy-irradiated mice. On Days 2, 3 and 5, the immobility of 0.5 Gy-irradiated groups was lower than that of the Sham-irradiated group; however, no significant differences were observed (Fig. 2).

### Effects of X-irradiation on antioxidants in the liver at 6 or 24 h after FST

**Fig. 3:**
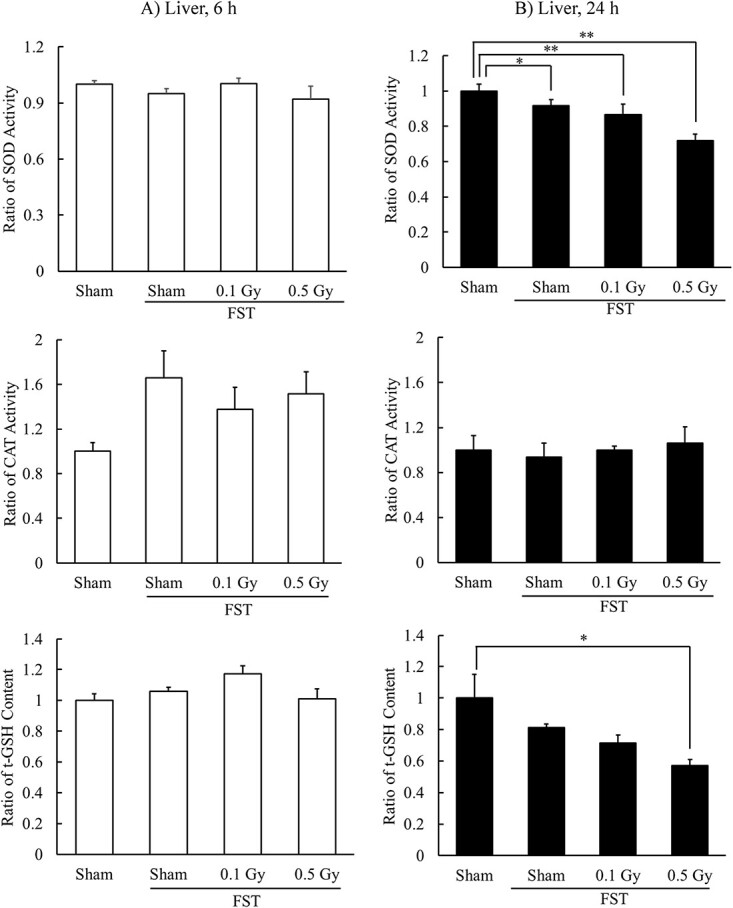
Effects of X-irradiation on antioxidants in mouse liver at 6 h (**A**) or 24 h (**B**) after FST. Mean ± standard error of the mean, *n* = 7, ^*^P < 0.05, ^*^^*^P < 0.01.

No significant changes were observed in liver antioxidant levels at 6 h after FST; however, CAT activity was increased (Fig. 3A).

In the FST group, SOD activity at 24 h after FST was significantly lower than that in the control (Sham [24 h]), and prior X-irradiation further decreased this activity. The t-GSH content in the 0.5 Gy + FST (24 h) group was significantly lower than that in the control group (Fig. 3B).

### Effects of X-irradiation on antioxidant contents in mouse livers with alcohol administration after FST

**Fig. 4:**
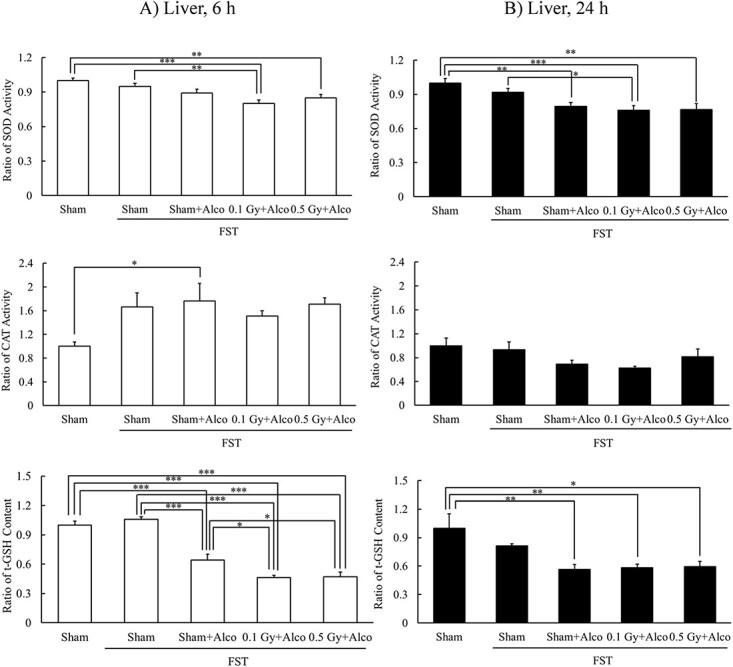
Effects of X-irradiation on antioxidants in mouse liver with alcohol administration at 6 h (**A**) or 24 h (**B**) after FST. Mean ± standard error of the mean, *n* = 4–7, ^*^P < 0.05, ^*^^*^P < 0.01, ^*^^*^^*^P < 0.001.

Antioxidant levels in the liver were analyzed 6 h after alcohol administration. CAT activity was significantly higher in the Sham + FST + Alco (6 h) group than that in the Sham (6 h) group, and t-GSH content was significantly lower than that in the Sham (6 h) and Sham + FST (6 h) groups. SOD activity was significantly lower in the 0.1 Gy + FST + Alco (6 h) group than that in the Sham (6 h) and Sham + FST (6 h) groups, and t-GSH content was significantly lower than that in the Sham (6 h), Sham + FST (6 h) and Sham + FST + Alco (6 h) groups. SOD activity was significantly lower in the 0.5 Gy + FST + Alco (6 h) group than that in the Sham (6 h) group, and the change in t-GSH content was similar to that in the 0.1 Gy + FST + Alco (6 h) group (Fig. 4A).

SOD activity was analyzed 24 h after alcohol administration. SOD activity was significantly lower in the Sham + FST + Alco (24 h), 0.1 Gy + FST + Alco (24 h) and 0.5 Gy + FST + Alco (24 h) groups than that in the Sham (24 h) group, and that in the 0.1 Gy + FST + Alco (24 h) group was significantly lower than that in the Sham + FST (24 h) group. T-GSH content was significantly lower in the alcohol-treated groups than in the control group (Fig. 4B).

### Effects of X-irradiation on hepatic functions in mouse sera with alcohol administration after FST

Previously reported data showed that the low-dose X-irradiation had no effect on hepatic function in normal mice [15]. Therefore, GOT and GPT activities in the 0.1 Gy + FST (6 or 24 h) and 0.5 Gy + FST (6 or 24 h) groups were not measured.

**Fig. 5:**
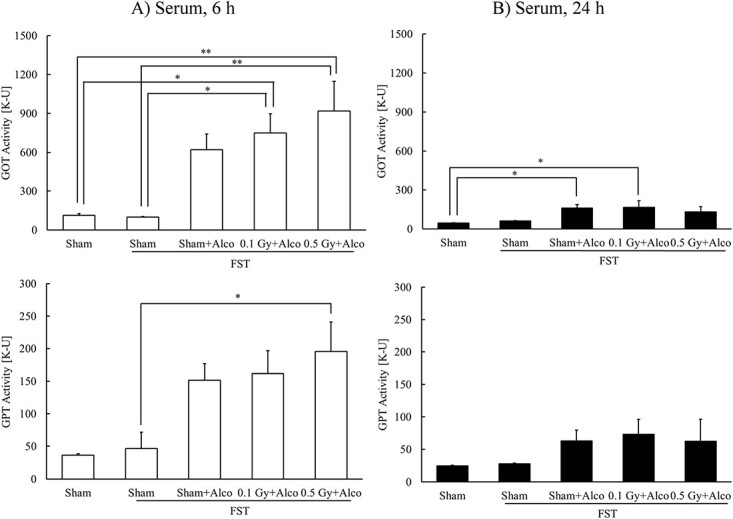
Effects of X-irradiation on hepatic functions in mouse serum with alcohol administration at 6 h (**A**) or 24 h (B) after FST. Mean ± standard error of the mean, *n* = 3–7, ^*^P < 0.05, ^*^^*^P < 0.01.

Serum GOT and GPT activities increased 6 h after alcohol administration following the FST. Moreover, GOT activity was significantly higher in the 0.1 Gy + FST + Alco (6 h) and 0.5 Gy + FST + Alco (6 h) groups than that in the Sham (6 h) and Sham + FST (6 h) groups, and GPT activity was significantly higher in the 0.5 Gy + FST + Alco (6 h) group than that in the Sham + FST (6 h) group (Fig. 5A).

GOT and GPT activities in the alcohol-treated groups 24 h after alcohol administration were lower than that at 6 h after alcohol administration. However, GOT activity in the Sham + FST + Alco (24 h) and 0.1 Gy + FST + Alco (24 h) groups was higher than that in the control group (Fig. 5B).

### Effects of X-irradiation on antioxidant activity in the brain, lungs and kidneys at 6 h after FST

**Fig. 6:**
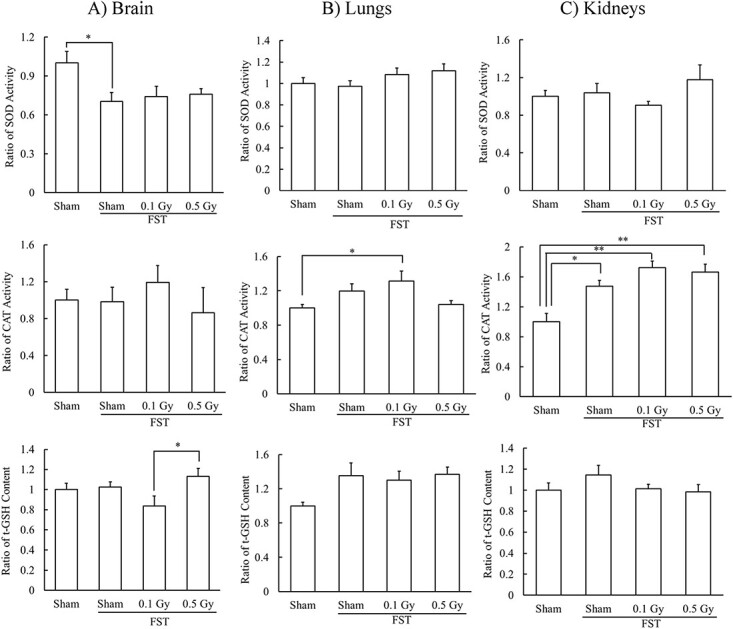
Effects of X-irradiation on antioxidants in mouse organs at 6 h after FST. Mean ± standard error of the mean, *n* = 5–7, ^*^P < 0.05, ^*^^*^P < 0.01.

In the brain, SOD activity was significantly lower in the Sham + FST (6 h) group than that in the Sham (6 h) group at 6 h after FST. However, that in the X-ray-irradiated groups was closer to that in the controls. Prior 0.5 Gy irradiation significantly increased the t-GSH content compared to prior 0.1 Gy irradiation (Fig. 6A).

In the lungs, CAT activity in the 0.1 Gy + FST (6 h) group was higher than that in the Sham (6 h) group; however, t-GSH content was slightly higher in the FST groups than that in the control group (Fig. 6B).

In the kidneys, CAT activity was significantly higher in the FST groups than that in the Sham (6 h) group and higher in the X-ray-irradiated groups than that in the Sham + FST (6 h) group (Fig. 6C).

## DISCUSSION

A bilateral causal relationship has been reported to exist between major depressive disorder (MDD) and alcohol use disorder (AUD) [16, 17]. In addition, following the earthquakes and the Fukushima nuclear power plant accident, some individuals developed depression and alcohol dependence due to anxiety related to the disaster and radiation exposure [18–24]. Although studies on the prevention of stress, alcohol consumption and depression in rodents in this regard have been conducted [25–30], no studies have evaluated the degree of forced high-dose alcohol administration-induced liver damage after FST for several days. Chronic stress from repeated FSTs has been reported to cause oxidative stress in the brain, as well as tissue degeneration and oxidative stress in peripheral tissues, including the liver, which is regulated by the brain [6, 7]. Thus, alcohol administration after FST would cause further oxidative stress in the liver. Although the results of our previous study also suggest that oxidative stress in the liver is increased by FST after low-dose irradiation [8], it is important to evaluate the risk of low-dose irradiation in this case. This study elucidates some of the effects of low-dose/high-dose-rate X-irradiation on complex disorders associated with these oxidative stressors, particularly with respect to liver injury.

A 5-day FST has been demonstrated to induce immobility (depression-like) in mice [14]. Therefore, mice were pre-irradiated with 3.0 mGy/h (dose rate)/0.5 Gy (total dose) and 0.6 mGy/h/0.1 Gy of γ-rays followed by FST for 5 days; the results revealed that the immobility state was suppressed on the second day of FST with 3.0 mGy/h (dose rate)/0.5 Gy (total dose) irradiation [8]. In addition, prior 1.2 Gy/m/0.1 Gy X-irradiation suppressed immobility on Day 5 of FST, and 1.2 Gy/m/0.5 Gy X-irradiation after five FSTs suppressed the immobility at the sixth FST [13]. However, in this study, the FST-induced immobility state tended to be worsened by pre-irradiation with 1.2 Gy/m/0.1 Gy of X-rays but suppressed by irradiation with 1.2 Gy/m/0.5 Gy of X-rays. The difference between the present results and those of our previous X-irradiation study may be related to the time of restraint to the cage during X-irradiation. In the previous study, restraint for ~2 min increased immobility as much as one round of FST [13]. In this study, although the restraint time was shorter (24 s), 0.1 Gy irradiation did not suppress the immobility. Based on these facts, it is difficult to say that prior high-dose-rate X-irradiation suppresses the FST-induced immobility. Therefore, the immobility suppression by pre-irradiation is dependent on low-dose-rate, not high-dose-rate.

Reactive oxygen species (ROS)-induced oxidative stress is also a key factor in the development of alcohol-induced hepatopathy [9–12]. Alcohol is metabolized to acetaldehyde primarily by alcohol dehydrogenase, cytochrome P450 2E1 and CAT [31], and acetaldehyde is metabolized to acetic acid by aldehyde dehydrogenase [32]. Metabolism by these enzymes produces ROS, such as hydroxyethyl, superoxide anion and hydroxyl radicals [31]. Thereafter, acetic acid promotes adenosine triphosphate (ATP) degradation and xanthine production, and malondialdehyde increases xanthine metabolism via xanthine oxidase [33]. In this study, alcohol administration decreased SOD activity and t-GSH content, increased GOT and GPT activity and caused liver damage. CAT activity in the liver was significantly increased after a period of 6 h, but not after 24 h, following alcohol administration, which might be attributed to alcohol metabolism. In addition, GOT and GPT activities significantly increased at 6 h after alcohol administration with 0.1 and 0.5 Gy irradiation and 0.5 Gy irradiation, respectively. However, 24 h after alcohol administration, liver function improved, although no significant difference was observed based on the presence or absence of irradiation or dose. In particular, 0.5 Gy irradiation temporarily worsened liver function, which recovered 24 h after alcohol administration. This result is consistent with the changes in t-GSH content in the liver. An important relationship exists between t-GSH content and alcohol metabolism; t-GSH content decreases because of the increased oxidative stress associated with its metabolism [34–37]. In our previous study, radon or thoron inhalation decreased t-GSH content in the liver 6 h after alcohol administration; however, t-GSH content increased 24 h after administration [38, 39]. Based on these findings, the content of t-GSH with a thiol (–SH) group, which exhibits a highly sensitive antioxidant effect, decreased 6 h after alcohol administration following FST because of its decomposition, which manifests acute liver function impairment. Prior X-irradiation decreased it to a greater degree; however, no significant change with or without X-irradiation was observed 24 h after administration, which indicated recovery from the impairment. In general, the amount of free radicals produced in the body upon 0.5 Gy irradiation is greater than that produced after 0.1 Gy irradiation. However, in this study, there was no significant difference between 0.1 and 0.5 Gy irradiation regarding the effects on liver injury. Although both types of irradiation showed early recovery from liver injury, the effects of 0.5 Gy irradiation may be greater than those of 0.1 Gy irradiation, probably because of the moderate stimulation induced by 0.5 Gy irradiation.

The degree of oxidative stress suppression caused by different irradiation dose rates is unclear. However, it has been demonstrated that the redox state of normal tissues differs depending on the organ [40]; the liver and kidneys have a high antioxidant function; the brain, pancreas and stomach have a low antioxidant function and low LPO levels; and the lungs, heart, small intestine, and large intestine have a low antioxidant function and high LPO levels. Given that antioxidant function enhancement using low-dose radiation is induced by small amounts of ROS [1, 41], the responses to various types of oxidative stress are hypothesized to be different. Furthermore, FST increases LPO levels in the rat brain, liver and kidneys [6] and decreases t-GSH content in the kidneys [42]. In a previous study, CAT activity and t-GSH content in the brain and kidneys decreased after FST for 5 days; however, they approached normal values with prior low-dose-rate irradiation. Pre-irradiation at 3.0 mGy/h (total dose of 0.5 Gy) combined with FST increased CAT activity in the lungs but decreased SOD activity and t-GSH content in the liver. These results suggest that the lungs are resistant to ROS, whereas the liver is susceptible [8]. Although SOD activity in the brain decreased following FST, prior high-dose-rate irradiation reduced the degree of its decrease, and prior high-dose-rate 0.5 Gy irradiation increased the t-GSH content. High-dose-rate X-irradiation in the previous study did not affect antioxidant functions in the brain after FST [13]. These differences probably were also influenced by the time of restraint during irradiation. After ~2 min of restraint, increased LPO levels and decreased t-GSH content were observed in the brain [13]. In this study, although the effects of restraint on antioxidant functions in the brain were not evaluated, it is considered to be less stressful than that determined in the previous study. In the lungs, 0.1 Gy prior irradiation increased CAT activity, and in the liver, the antioxidant function was maintained even with prior irradiation and FST. Pre-irradiation increased CAT activity in the kidneys. These findings suggest that the effects of high-dose-rate/low-dose-rate pre-irradiation on each organ after FST vary and that the lungs are resistant to oxidative stress.

The FST-induced immobility state is particularly related to oxidative stress in the brain [6, 7, 43], and improving it suppresses immobility [44]. In this study, prior high-dose-rate 0.5 Gy irradiation was found to reduce oxidative stress in the brain after FST; however, we could not suggest that the effects of this irradiation inhibited immobility. However, the antioxidant function in the liver decreased 24 h, not 6 h, after the irradiation and FST, suggesting that the effects of low-dose/high-dose-rate irradiation occur relatively late. Therefore, experiments with more frequent FSTs may clarify the effects of radiation on the brain’s antioxidant functions and immobility.

This study had some limitations. First, given that this study did not evaluate the effects of high-dose irradiation, dose-dependency effects were not demonstrated. Second, as this was an *in vivo* study, the underlying mechanisms could not be analyzed in detail. Third, psychological stress parameters, such as noradrenaline and cortisol, were not measured; therefore, the degree of low-dose irradiation, FST or alcohol administration-induced psychological stress was not adequately assessed. Fourth, the results of this study provide insight into the effects of low-dose-rate acute exposure but do not contribute to elucidating the medium- to long-term effects of chronic exposure. Finally, the mouse model in this study was not that of MDD or AUD, and the direct effects of irradiation on those diseases could not be elucidated.

## CONCLUSION

Low-dose/high-dose-rate irradiation did not suppress FST-induced immobility. However, low-dose irradiation increased t-GSH content (enhanced antioxidant function) 24 h after FST and alcohol administration compared with that seen 6 h after administration, suggesting its contribution to liver function improvement. Furthermore, high-dose-rate irradiation was suggested to cause different oxidative effects in different mouse organs, and its effects were suggested to differ from those of low-dose-rate irradiation. Further investigation of the effects of low-dose irradiation on mental health caused by combined environmental oxidative stressors is required. Moreover, the effects of other exposure methods and α-ray irradiation should be examined using histopathological diagnostics and immunohistochemical analyses to clarify the issues mentioned above.

## CONFLICT OF INTEREST STATEMENT

The authors declare that they have no conflicts of interest.

## FUNDING

This work was supported by JSPS KAKENHI (grant number JP 21 K04943).
